# The Legal Position in Maternity Cases: The Law, the Nurse, and the Compromise

**Published:** 1914-08-29

**Authors:** 


### THE LEGAL POSITION IN MATERNITY CASES.

### The Law, the Nurse, and the Compromise.

A question about which practitioners are often consulted is the legal position of a maternity nurse who is engaged to attend a confinement on a particular date, and the confinement does not take place until either some time before or some time after such date. The amount involved in such cases is usually of so small a character as to preclude the question being taken to the High Court of Justice for determination, and consequently the nursing profession is without a pronouncement of the High Court on the question, and the legal principles have been left for enunciation to the judges of the county courts. In laying down the principles which govern these cases much assistance is to be derived from the judgment of his Honour Judge Lumley-Smith in an action that was heard by him in the City of London Court in December 1912. A maternity nurse should always be careful to see that she is engaged for a particular date, and not for "a confinement when it shall take place," and these observations are written upon the assumption that the nurse has adopted this precaution. As a first principle it may be laid down that if a nurse is engaged to attend a confinement on a particular date at a remuneration, then contractual relations are established and a contract is concluded, and so long as the nurse presents herself on that date with a view to performing her part of the contract, she has done all that is necessary for her to do in order to fulfil the contract, and it is immaterial whether the confinement has already taken place. Let us illustrate the legal position by a concrete example.

A nurse engages herself for maternity cases on April 1, May 1, June 1, and July 1, and she successfully fulfils her engagements for April 1 and May 1. She then presents herself on June 1 in order to fulfil her engagement booked for that date. Whether or not her services are then required is immaterial, for she has done all that is in her power to fulfil her part of the contract. If the confinement took place prior to the date arranged between the parties, then, quite apart from the fact that the contract was for a particular date, the nurse could not have attended, since she was engaged with her May case, while, if the confinement was not expected until July, again the nurse could not attend, because she was already booked for July. As has been stated, the nurse is secure in her position in that the contract was for a particular date, and these observations are appended by way of parenthesis to illustrate the soundness and wisdom of the law governing such cases, for otherwise a nurse could never safely book engagements except at long intervals, which would seriously militate against nurses earning their livelihood at nursing. It must, however, be remembered that the nurse must always present herself on the date of her engagement, or write to inquire whether her services are required, notwithstanding that she may possess the knowledge that the confinement has already taken place, for even in such a case it might be that the employer had temporarily engaged another nurse until the date when the original nurse was expected. If, therefore, the nurse duly presents herself on the agreed date, thus affording evidence of her willingness to fulfil her part of the contract, and she is prevented from so doing by the circumstances already discussed, she is entitled to treat the contract as broken, and to sue her employer for damages for breach of contract, the measure of such damages being her fee and her board and lodging during the period for which she was engaged.

There is, however, a disinclination among nurses to insist on the full rights to which the letter of the law strictly entitles them. As a general rule, a compromise is effected, by which the nurse receives half the fees she would have earned. This arrangement leaves her free to accept, if she can get it, another case in place of that of which unforeseen events have deprived her. If she does obtain such a case, she scores by being paid one and a half times over for her work; if she does not, at least she gets a welcome rest and holiday, with half-pay to carry her through it. Another, but less frequent, method of composition is for the nurse to receive her full fees, but to refund them in respect of any period during which she obtains other remunerated work. This plan has little to recommend it, and it is mentioned merely because it is occasionally adopted.

It may be asked: Why should a nurse accept less than her legal rights? Why shouldn't she insist on full fees when she is entitled to them in law? The answer is very simple. Maternity nurses, like doctors, depend on the good will and the recommendation of their satisfied patients for future introductions. Indeed, nurses are doubly dependent on others, for they have to please not only the patients, but the doctors too. Nurses who refused all compromise, and stickled for full fees whenever their patients' confinements happened later or earlier than expected, would soon find themselves losing their practice. So that even from the purely selfish pecuniary aspect, it is to their advantage to forego their legal rights. Further, they naturally have to bear in mind the "patients' point of view." The father and mother expectant are not to blame if a baby arrives at some date rather distant from that which was predicted. They have in any case the worry of securing a substitute nurse at a moment's notice, and, of course, they have to pay her full fees. To be called upon for the same amount by the previously engaged nurse, who does, in fact, do nothing to earn it, is rather hard lines. So that the half-fees compromise is defensible from the practical standpoint as a fair and reasonable arrangement, although less than her legal rights.

